# CT Radiomics and Machine Learning for Three-Class Bone Density Classification: An Exploratory Comparison Across Lumbar Vertebral Levels

**DOI:** 10.3390/tomography12100147

**Published:** 2026-10-02

**Authors:** Asiye Sozeri, Halitcan Batur, Nurdan Cay

**Affiliations:** 1 Department of Radiology, Karabuk Training and Research Hospital, 5000 Evler Bahcelievler Mah., 78020 Karabuk, Turkey; 2 Department of Radiology, Falu Lasarett, 791 82 Falun, Sweden; 3 Department of Radiology, Aspetar Orthopaedic and Sports Medicine Hospital, Doha P.O. Box 29222, Qatar

**Keywords:** tomography, DEXA scan, radiomics, bone mineral density, osteoporosis, osteopenia, machine learning

## Abstract

**Objectives:** The aim of this study is to provide early diagnosis of osteoporosis, osteopenia, and normal bone mineral density (BMD) on CT scans utilizing machine learning and radiomics techniques. **Methods:** In this retrospective study, 874 patients who met the predefined eligibility criteria were included. BMD was categorized as normal, osteopenia, or osteoporosis according to DEXA-derived T-scores. Lumbar vertebral bodies were automatically segmented on abdominal and lumbar CT images, and radiomic features were extracted from the segmented regions. Logistic regression (LR), decision table (DT), random forest (RF), multilayer perceptron (MLP), and bagging models were developed to distinguish among the three BMD categories. Model performance was evaluated using accuracy, F-score, area under the receiver operating characteristic curve (ROC area), and area under the precision–recall curve (PRC area). **Results:** Notably, the best results were observed in the L3 vertebra among the 874 patients included in the study. Accordingly, the MLP and LR methods showed the greatest accuracy value of 83.5%, F-score value of 81.2%, ROC area value of 0.912, and PRC area value of 0.892 in cases with normal BMD. At the L2 vertebral level, the LR approach produced the greatest results for patients with osteoporosis (Acc: 72.5%, F-score: 72.5%, ROC area: 0.897, and PRC area: 0.797). **Conclusions:** In the three-class BMD assessment, the models showed the highest performance for normal BMD and the lowest performance for osteopenia. Among the individual lumbar levels, L3 demonstrated the highest radiomics-based classification performance, whereas L1 showed comparatively lower performance. These level-specific findings should be regarded as exploratory and should not be interpreted as supporting clinical BMD classification from a single vertebra. CT radiomics may have potential as an opportunistic screening tool to identify patients who may benefit from formal DEXA or further osteoporosis evaluation; however, patient-level multivertebral validation is required before clinical implementation.

## Simple Summary

Osteoporosis and osteopenia weaken the bones and increase the risk of fractures, but they may remain undetected until a fracture occurs. Bone density is usually measured with a dedicated scan; however, many patients already undergo computed tomography scans for other medical reasons. In this study, we examined whether routine computed tomography images could also provide useful information about bone health. Lumbar spine images from 874 patients were automatically analyzed, and several machine learning methods were used to classify patients as having normal bone density, osteopenia, or osteoporosis. The most successful results were obtained from the third lumbar vertebra, particularly for identifying normal bone density. Osteopenia was the most difficult group to classify. These findings suggest that information obtained from existing computed tomography scans could help identify patients who may need formal bone density testing. Further studies using multiple vertebrae and independent patient groups are needed before this method can be introduced into clinical practice.

## 1. Introduction

Osteoporosis is characterized by reduced bone mass and structural deterioration of bone tissue, resulting in increased fragility and a higher risk of fractures [1]. Osteopenia, an early indicator of osteoporosis, reflects decreased bone mineral density (BMD). With the aging population, the prevalence of osteoporosis and related fractures is steadily rising, making it the most common skeletal disorder among the elderly and a major public health concern with substantial economic impact. According to the International Osteoporosis Foundation, over 40% of middle-aged women are expected to experience at least one osteoporotic fracture during their lifetime. The proximal femur, vertebra, and distal radius represent the most clinically relevant fracture sites, with hip fractures posing the most severe complication due to their high morbidity and one-year mortality rates [2]. While various therapeutic options and their associated adverse effects are still under investigation, emphasis should be placed on early detection and diagnosis of osteoporosis and osteopenia rather than solely on treatment. Dual-energy X-ray absorptiometry (DEXA) remains the gold standard for diagnosis; however, routine chest and abdominal computed tomography (CT) scans performed for other clinical purposes provide a unique opportunity for opportunistic osteoporosis screening, enabling improved detection without additional cost, time, or radiation exposure [3]. The integration of artificial intelligence (AI) into such opportunistic screening approaches may further enhance their diagnostic value. Radiomic features—capturing information from pixel intensity and spatial organization to tissue density and wavelet characteristics—when combined with machine learning (ML) techniques, can offer a comprehensive evaluation of bone quantity and quality.

The aim of this study was to compare the radiomics-based discriminative performance of individual lumbar vertebral levels for identifying CT patterns associated with normal BMD, osteopenia, and osteoporosis, using level-specific DEXA measurements as the reference. The study was designed as an exploratory vertebral-level analysis and was not intended to replace standard patient-level DEXA classification based on the combined L1–L4 assessment.

## 2. Materials and Methods

### 2.1. Ethics Approval and Consent to Participate

This retrospective study was approved by the Ethics Committee of our institution (Approval No: E2-23-4717, dated 16 August 2023). The study was conducted in accordance with the Declaration of Helsinki and its later amendments. Due to the retrospective nature of the study, the requirement for informed consent was waived by the Ethics Committee.

### 2.2. Patient Consent for Publication

Not applicable. No identifiable patient information or identifiable images are included in this manuscript.

### 2.3. Data Collection

In this retrospective study, patients who underwent both DEXA and CT examinations between February 2019 and September 2023 were reviewed. Demographic information, known chronic diseases, prescribed or previously recorded medications, and surgical histories were retrieved and recorded from the hospital’s electronic medical record system.

Exclusion criteria were identified through a combined review of electronic medical records, radiology reports, and CT images. Demographic characteristics, comorbidities, medication use, previous radiotherapy or chemotherapy, history of malignancy, neurological and endocrine disorders, organ transplantation candidacy, and previous spinal surgery were determined from the electronic medical records. Vertebral fractures, vertebral cement augmentation, malignant vertebral lesions, bone metastases, severe or marked rotoscoliosis, and other structural spinal abnormalities were assessed through radiology report review and direct evaluation of the CT images.

All abdominal and lumbar CT examinations were reviewed by a radiologist with 6 years of experience in general radiology. Axial images and multiplanar sagittal and coronal reformations were evaluated according to a standardized review protocol. The review included assessment of vertebral morphology, cortical disruption, vertebral height loss, endplate deformity, cement augmentation, postoperative changes, focal malignant lesions, metastatic involvement, and marked spinal deformity. Equivocal cases were discussed with a senior radiologist and resolved by consensus. Because the initial image assessment was performed by a single radiologist, formal interobserver agreement was not evaluated.

A lumbar vertebral fracture was considered present when a previous fracture was documented in the electronic medical record or radiology report, or when imaging findings consistent with a vertebral compression fracture were identified during direct CT review. Imaging findings suggestive of vertebral fracture included vertebral height loss, endplate deformity, cortical disruption, and anterior or lateral wedging. Patients with either acute or chronic lumbar vertebral fractures were excluded because vertebral deformity and altered trabecular architecture could affect both DEXA measurements and radiomic features.

Patients with previous lumbar spinal surgery or vertebral cement augmentation were excluded because metallic hardware, postoperative alterations, and cement-related artifacts could interfere with automated segmentation and radiomic feature extraction. Patients with malignant vertebral lesions, known bone metastases, or previous bone-targeted radiotherapy or chemotherapy were excluded because these conditions and treatments may alter vertebral structure and BMD independently of primary osteoporosis. Severe or marked rotoscoliosis was an exclusion criterion because vertebral rotation and deformity could impair reliable segmentation and introduce variability into radiomic measurements. CT examinations of insufficient quality because of severe motion, beam-hardening, or other artifacts that prevented reliable radiologic assessment or segmentation were also excluded.

Patients using medications known to affect bone metabolism or increase the risk of low BMD, including corticosteroids, aromatase inhibitors, selective serotonin reuptake inhibitors, and antiepileptic drugs, were excluded to reduce treatment-related confounding. Patients with endocrine disorders, including hyperthyroidism, hypothyroidism, thyroid masses or lesions, and diabetes mellitus, were excluded because these conditions may affect bone turnover and BMD independently of age-related or postmenopausal bone loss. Patients with neurological diseases, including Parkinson’s disease and Alzheimer’s disease, were excluded because reduced mobility, increased fall risk, impaired nutritional status, and disease-related functional limitations may independently affect bone health. Candidates for organ transplantation were excluded because advanced organ dysfunction, associated metabolic abnormalities, and current or anticipated immunosuppressive treatment may substantially alter bone metabolism. Patients with HIV/HCV coinfection were excluded because chronic infection and its associated treatment may also affect BMD.

A total of 10,169 patients underwent both DEXA and abdominal or lumbar CT during the study period. Of these, 6407 were excluded because the interval between DEXA and CT exceeded 3 months. Among the remaining 3762 patients, 2888 were excluded according to the predefined exclusion criteria. To avoid duplicate counting, each excluded patient was assigned to a single primary exclusion category. Consequently, 874 patients were included in the final analysis. The final cohort comprised 421 contrast-enhanced and 453 non-contrast CT examinations. The patient selection process is presented in Figure 1.

### 2.4. DEXA and CT Acquisition Protocol

DEXA scans were performed using a Lunar Bone Densitometer (Lunar iDXA GE HealthCare, Madison, WI, USA) T-scores and BMD (g/cm^2^) values were obtained separately for the L1 to L4 lumbar vertebrae. For each patient, individual T-scores were recorded for the L1, L2, L3, and L4 vertebral bodies. Contrast-enhanced and non-contrast abdominal and lumbar CT examinations were acquired at the Radiology Department of our center using a 128-slice multidetector CT scanner (Revolution Evo 128, GE Healthcare, Milwaukee, WI, USA). Scans were obtained in the supine position, from the diaphragmatic dome to the inferior margin of the symphysis pubis, using dual scout topograms for localization. The acquisition protocol included the following parameters: collimation, 2 mm; rotation time, 0.6 s; pitch, 1; field of view, 40 cm; tube voltage, 100–120 kV; tube current, 200–400 mA; slice thickness, 1.25 mm; and reconstruction matrix, 512 × 512. For contrast-enhanced CT scans, an iodinated contrast agent was administered via an automatic power injector at a rate of 3 mL/s, and images were acquired during the portal venous phase (between 65 and 70 s after injection).

### 2.5. Vertebral Selection, Segmentation, Preprocessing, and Radiomic Feature Extraction

CT images were retrieved from the hospital Picture Archiving and Communication System (PACS) in Digital Imaging and Communications in Medicine (DICOM) format. The open-source software 3D Slicer (version 5.2.2; www.slicer.org, accessed on 15 October 2024) was used for segmentation, and the PyRadiomics Python 3.9 library was used to extract radiomic features. For automatic segmentation, we used the Total Segmentator plugin within the 3D Slicer software [4]. All automated vertebral segmentations were subsequently reviewed visually by a radiologist for anatomical accuracy across the entire cohort (874 cases).

For all cases, the preprocessing parameters were standardized: under the “resampling and filtering” module, the bin width was set to 64, and voxel resampling was performed with an isotropic resolution of 1 × 1 × 1 mm^3^. Using automated segmentation (Figure 2), a total of 93 radiomic features were extracted from each vertebra, including 18 first-order and 75 texture-based features. These features comprised gray-level dependence matrix (GLDM), gray-level co-occurrence matrix (GLCM), first-order, gray-level run length matrix (GLRLM), gray-level size zone matrix (GLSZM), and neighborhood gray tone difference matrix (NGTDM) parameters. For each vertebra (L1–L4), the extracted features included:

- 18 first-order features;
- 24 GLCM-based second-order features;
- 14 GLDM-based second-order features;
- 16 GLRLM-based higher-order features;
- 16 GLSZM-based higher-order features;
- 5 NGTDM-based higher-order features, resulting in a total of 93 features. A complete list of the 93 extracted radiomic features, itemized by category, is provided in Supplementary Table S1. Each feature was evaluated separately for all lumbar vertebrae across the dataset.

### 2.6. Statistical Analysis

All statistical analyses were performed using SPSS software (version 11.5; IBM Corp., Armonk, NY, USA). Descriptive statistics for quantitative variables were expressed as mean ± standard deviation (SD) or median (minimum–maximum), while categorical variables were presented as counts and percentages [n (%)]. The chi-square (χ^2^) test was used to examine associations between categorical variables. A *p*-value < 0.05 was considered statistically significant.

All ML analyses were performed in R (version 4.5.1; R Foundation for Statistical Computing, Vienna, Austria) using the RWeka (version 0.4-46) and e1071 (version 1.7-16) packages. Feature selection was carried out using Correlation-based Attribute Evaluation within the training folds only to avoid information leakage. The selected features were then used to train supervised classifiers including Logistic Regression (LR), Multilayer Perceptron (MLP), Bagging, Decision Table (DT), and Random Forest (RF). Model performance was assessed via stratified 10-fold cross-validation, and the complete cross-validation workflow was repeated 1000 times using different random fold assignments to ensure robustness. Predictive performance was reported as the mean (and variability) of accuracy (Acc), F-score, area under the ROC curve (ROC area), and area under the precision–recall curve (PRC area) across all repetitions. All modeling steps (preprocessing, feature selection, training, and evaluation) were executed under a strictly nested workflow to prevent data leakage. The analysis code and parameter settings were preserved to enable full reproducibility of the cross-validation and repeated resampling procedure. As the repetition-level outputs of the original 1000-repetition cross-validation were not retained for SD reporting, an independent reanalysis of the patient-level dataset was performed in Python (scikit-learn 1.3.2) as a reproducibility check, following the same overall workflow with 100 repetitions of stratified 10-fold cross-validation; mean ± SD results from this check are reported in Supplementary Table S2.

## 3. Results

The study included 874 patients with a mean age of 64.36 ± 9.73 years. Among them, 674 (77.1%) were female and 200 (22.9%) were male (Table 1).

A statistically significant difference was observed in the T-score-based classifications among the L1, L2, L3, and L4 vertebrae (*p* < 0.001). According to the L4 vertebra, 20.4% of patients were classified as having osteoporosis, compared with 15.9% at L3, 30.3% at L2, and 21.7% at L1. For osteopenia, the proportions were 37.3% (L4), 40.4% (L3), 38.0% (L2), and 46.0% (L1). Patients classified as normal BMD were 42.3%, 43.7%, 31.7%, and 32.3% for L4, L3, L2, and L1, respectively (Table 2).

### 3.1. Model Performance for the L4 Vertebra

For radiomic features extracted from the L4 vertebra, Correlation Attribute Evaluation was used to rank feature importance and to quantify the contribution of individual variables to the outcome (Figure 3). In total, 13 variables (12 independent and 1 dependent) were included in the general model, and all ML analyses were performed using these features.

For the L4 vertebra, five ML algorithms—LR, MLP, Bagging, DT, and RF—were ap- plied to evaluate predictive performance. Based on Acc and F-score, the best overall performance was achieved by the RF method. The RF model yielded an Acc of 64.3%, indicating that the model correctly predicted the class (normal, osteopenia, or osteoporosis) in 64.3% of cases. Specifically, prediction accuracies for the normal, osteopenic, and osteoporotic groups were 73.4%, 60.4%, and 52.2%, respectively (Table 3).

### 3.2. Model Performance for the L3 Vertebra

For radiomic features extracted from the L3 vertebra, Correlation Attribute Evaluation was applied to assess feature importance and the impact of individual variables on the outcome (Figure 4). According to the results of the feature importance test and clinical relevance assessment, the final model included the following variables: firstorder Total Energy, glrlm Gray Level Non-Uniformity Normalized, firstorder Median, firstorder Interquartile Range, firstorder Uniformity, ngtdm Contrast, glszm Gray Level Non-Uniformity Normalized, firstorder 10th Percentile, glcm Id, glcm Imc2, gldm Small Dependence Emphasis, and glszm Zone Percentage. In total, 13 variables (12 independent and 1 dependent) were included in the general model, and all ML analyses were performed using these features.

For the L3 vertebra, based on Acc and F-score, the LR model demonstrated the best overall performance. The LR-based model achieved an accuracy of 71.6%, indicating that the classification of normal, osteopenic, and osteoporotic patients was correct in 71.6% of cases. Specifically, the model correctly classified 80.4% of normal, 72.2% of osteopenic, and 46.0% of osteoporotic patients (Table 4).

### 3.3. Model Performance for the L2 Vertebra

Correlation Attribute Evaluation was applied to L2-derived radiomic features to identify the most informative variables and assess their impact on the outcome (Figure 5). In total, 13 variables (12 independent and 1 dependent) were included in the general model, and all ML analyses were performed using these features.

For the L2 vertebra, the LR model achieved the highest overall performance, as reflected by both Acc and F-score. The LR model achieved an Acc of 70.4%, indicating that the classification of normal, osteopenic, and osteoporotic patients was correct in 70.4% of cases. Specifically, 75.8% of normal, 64.2% of osteopenic, and 72.5% of osteoporotic patients were correctly predicted (Table 5).

### 3.4. Model Performance for the L1 Vertebra

Radiomic features derived from the L1 vertebra were evaluated using the Correlation Attribute Evaluation method to determine feature importance and to examine the effect of each feature on the outcome (Figure 6).

For the L1 vertebra, the LR model yielded an accuracy of 68.1%, indicating that the classification of normal, osteopenic, and osteoporotic patients was correct in 68.1% of cases. Specifically, the model correctly classified 70.9% of normal, 75.4% of osteopenic, and 48.4% of osteoporotic patients (Table 6).

The highest and lowest model performance results for each vertebral level are summarized in Table 7. The mean ± SD variability estimates from the reproducibility analysis are provided in Supplementary Table S2.

According to our findings, in the overall population, the lowest values for accuracy, F-score, ROC area, and PRC area were obtained with the RF, Bagging and DT methods for the L1 vertebra, whereas the highest values were achieved with the LR method for the L3 vertebra. Interestingly, among osteoporotic patients, the L3 vertebra showed the lowest accuracy, F-score, and PRC area, while the best performance in this group was obtained at the L2 vertebral level. Among the ML algorithms, the LR model demonstrated superior results across osteopenic, osteoporotic, and overall patient groups. Additionally, the MLP method yielded slightly higher Acc and F-score values than LR in patients with normal BMD. The lowest performance across all metrics in normal BMD patients was observed with the DT method, whereas RF and Bagging generally produced lower results in other subgroups.

## 4. Discussion

In the present study, CT-derived radiomic features and ML methods were used to perform a three-class BMD assessment separately at the L1, L2, L3, and L4 vertebral levels. The purpose of these level-specific analyses was to compare the relative radiomic information and discriminative performance of individual lumbar vertebrae. Importantly, these analyses were exploratory and were not intended to support clinical BMD classification from a single vertebral body. In routine clinical practice, lumbar spine BMD interpretation is based on the combined assessment of all evaluable vertebrae from L1 to L4 rather than on an isolated vertebral level.

At L3, where the proportion of normal BMD was highest (43.7%), MLP achieved the highest Acc and F-score for normal BMD, whereas LR performed best for most other metrics. For osteopenia, Acc and F-score were highest at L1, where osteopenic cases were most frequent, while ROC and PRC areas were higher at L3, possibly reflecting the similar proportion of osteopenic cases at these two levels (>40%). For osteoporosis, ROC area exceeded 0.835 in all five models, with the best discrimination at L2, whereas Acc and F-score at L3 remained below 50%, likely reflecting the low proportion of osteoporotic cases at this level (15.9%).

Many patients presenting to the outpatient clinic with back pain complaints typically undergo lumbar CT scans rather than DEXA scans, which are used to monitor symptoms such as lumbar disc herniation and lumbar spondylolisthesis [5]. Therefore, in the absence of DEXA, there is a need for reliable diagnostic tools to screen for osteoporosis in the elderly, prevent complications, and develop a diagnosis and treatment plan as soon as possible [6]. For these purposes, opportunistic CT images, radiomics, and ML models were used in our study.

Defining regions of interest (ROIs) is the most important part of radiomics analysis, as subsequent predictions are based on these defined regions [7]. In manual ROI segmentation, data may vary from person to person. Compared to manual and semi-automatic segmentation methods, automatic segmentation is considered more effective and reproducible in radiomics analysis [8]. We performed our measurements using automatic segmentation on the same software to achieve more effective, reproducible results and significantly reduce the possibility of subjective outcomes.

Opportunistic CT scans are a term used to describe diagnostic CT imaging obtained for medical evaluation, particularly not for the purpose of assessing bone health, and subsequently used to estimate BMD [9]. In CT scan measurements, the Hounsfield unit (HU) values are obtained that do not include cortical bone, which leads to a significant lack of information and may not accurately reflect bone density [10]. In a study conducted using opportunistic CT, an HU value > 160 is associated with a low probability of osteoporosis on DEXA and therefore does not necessitate further screening unless a vertebral fracture is detected. However, for HU ≤ 110, the likelihood of osteoporotic or osteopenic BMD results is high, and therefore further evaluation may be necessary for bone health. If the HU value is between 110 and 160, there is a moderate risk of abnormal BMD [11]. In another study, it was found that HU values obtained from CT showed moderate agreement with BMD in DEXA studies [12]. Therefore, it is suggested that HU values obtained from lumbar CT scans can be used in the diagnosis of osteoporosis [13]. Beyond mean attenuation, radiomic features capture textural information of trabecular bone, although their robustness to acquisition and reconstruction parameters requires careful consideration [14]. Our study focused on the lumbar vertebrae, which are also specifically evaluated in DEXA, and aimed to assess BMD using radiomics and ML methods rather than HU values obtained from opportunistic CT.

Due to the possibility that intravenous contrast medium (IVCM) may enter the vertebral trabecular area and increase attenuation at this level, a study examining the effect of IVCM use on HU measurements at the L1 vertebra demonstrated that this effect was minimal in a middle-aged and elderly population; this is an age group in which opportunistic CT-based screening is likely to be used [15]. Another study emphasized that the application of IVCM has no effect on vertebral body attenuation or on the diagnosis of osteoporosis by DEXA [3]. Therefore, in our study, both contrast-enhanced and non-contrast-enhanced CT scans were included, as we considered that they would not have a significant effect on the results.

Over the past few years, studies have been reported using tissue characteristics extracted from DEXA/magnetic resonance imaging/X-ray for the detection, diagnosis, and classification of osteoporosis [16,17,18]. However, the predicted performance in osteoporosis diagnosis remains inadequate, and the reported AUC for osteoporosis classification is approximately 0.8. This situation may be attributed to the fact that the selected ROIs are not three-dimensional reconstructions of all vertebral bodies, which may lead to the loss of some relevant information and affect the accuracy of the prediction model. Further research is needed to improve prediction performance. CT images have been converted into volumetric data, which is more suitable for 3D radiomics feature analysis of trabecular bone. In the literature, studies on CT radiomics for osteopenia–osteoporosis and normal BMD classification are insufficient. In this study, we performed a three-way classification (normal–osteopenia–osteoporosis) in BMD assessment based on radiomics analyses of CT images and defined it using ML methods.

Various studies [19,20] have confirmed that the lumbar vertebrae are a good observation area for bone loss in all skeletal structures. Among the lumbar vertebrae, L3 has the highest specificity [19], as L3 has been found to be the vertebral body with the highest bone metabolism rate and lowest bone density among all vertebral bodies. Studies have been shown to use the L3 vertebra to extract radiomics signatures and significantly distinguish osteopenia from osteoporosis (AUC = 0.96) [7]. Among the evaluated levels in our study, L3 showed the highest overall radiomics-based classification performance, whereas L2 showed favorable performance in the differentiation of osteoporosis. L1 demonstrated comparatively lower performance. These findings suggest that radiomic information may vary across lumbar vertebral levels. These level-specific differences should be regarded as hypothesis-generating rather than as a basis for single-vertebra clinical classification. Among the ML methods evaluated, LR provided favorable overall performance; however, this finding also requires validation in independent datasets.

In the study by Zhihao Xue and colleagues [21], the time interval between lumbar CT and DEXA examinations was not considered, leaving the evaluation of results incomplete. In the study by Shigeng Wang and colleagues [22], similarly to our study, it was assumed that the condition of the bone remained relatively stable within 3 months, and the study was initiated based on the 3-month scale between CT and DEXA values. This assumption is grounded in the physiology of bone remodeling, in which a full remodeling cycle typically spans several months; clinically meaningful changes in BMD are therefore not expected within a 3-month window in patients without acute metabolic, endocrine, or pharmacological perturbations, which were themselves exclusion criteria in the present study. The aim of the study by Yan-Wei Jiang and colleagues [23] was to evaluate the effectiveness of osteoporosis screening using a radiomics signature model based on routine preoperative lumbar vertebral CT scans. Ninety-nine patients with lumbar vertebral CT and DEXA scans within 14 days were included in the study, and the lumbar vertebrae were divided into two groups: osteoporosis and non-osteoporosis. The free and open-source 3D Slicer (https://www.slicer.org) was used for 3D segmentation of the vertebral bodies. The images were semi-automatically segmented in the vertebral areas, and these parts were removed to reduce the effect of the vertebral pedicles. In this study, the sample size was small, no age limit was set, and the ML method was not used. In contrast, the present study included a larger population of patients aged over 50 years and applied multiple ML methods to distinguish normal BMD, osteopenia, and osteoporosis. Automated segmentation was performed and vertebral levels were analyzed separately.

In the study by Baisen Chen et al. [6], radiological features were extracted from CT images of an elderly population (≥60 years old) consisting of a small sample (182 patients) who had been admitted to hospital for surgical treatment and had undergone routine pre- operative lumbar CT and DEXA examinations within one week, in order to evaluate the effectiveness of the radiological model in the diagnosis of osteoporosis. The 3DSlicer soft- ware was used to provide semi-automatic segmentation (vertebral pedicles removed). Three different classifiers (RF, SVM, KNN) were used to create the ML model. The AUC values for osteoporotic cases in the test sets for all three models (RF, SVM, KNN) were found to be 0.826, 0.838, and 0.796, respectively. When considering the ROC area values of osteoporotic cases in the ML models in our study, taking all lumbar vertebra levels into account, the ROC area values in the five models were found to be higher in the range of 0.835–0.897 compared to the smaller number of ML models by Baisen Chen and colleagues [6]. We found the best result in osteoporotic cases at the L2 vertebral level with the LR method (0.897).

Studies with a two-label classification (normal and abnormal BMD) generally achieved relatively higher performance compared to studies with a three-label classification (normal, osteopenia, and osteoporosis) [24]. Distinguishing normal BMD, osteopenia, and osteoporosis is inherently more challenging, particularly because osteopenia represents an intermediate category with overlapping imaging characteristics. In the present study, the highest performance was observed for normal BMD, whereas osteopenia was the most difficult category to classify. The use of multiple ML algorithms and 10-fold cross-validation allowed comparison of model performance across vertebral levels and classifiers.

After extraction of 93 radiomic features from each vertebral level, first-order Total Energy was retained as the principal variable for the ML analyses. This finding suggests that global voxel-intensity information may contribute substantially to BMD-related classification in the present dataset. However, dependence on a limited number of selected features may also restrict model generalizability.

The intended clinical role of the proposed approach is opportunistic screening and triage rather than replacement of DEXA. The model may potentially be applied to abdominal or lumbar CT examinations obtained for other clinical indications to identify patients with imaging patterns associated with low BMD who may benefit from formal DEXA, fracture-risk assessment, or further osteoporosis evaluation. A CT-based model output should therefore be interpreted as a screening signal rather than as a definitive diagnosis. Final BMD classification and treatment decisions should continue to rely on established clinical assessment and standard DEXA interpretation.

This study has several notable strengths. The cohort was relatively large (874 patients) compared with most prior CT-radiomics studies of bone density, which have typically included fewer than 200 patients. All CT examinations were acquired using a single, standardized scanner protocol, which reduced acquisition-related variability. Model performance was assessed using repeated stratified 10-fold cross-validation across 1000 repetitions, and multiple machine learning algorithms were compared, providing a robust internal evaluation framework.

Several limitations should be acknowledged. First, model performance was evaluated separately for each vertebral level using level-specific DEXA measurements, whereas clinical lumbar BMD classification relies on the combined L1–L4 assessment; the present findings therefore represent a methodological comparison among vertebral levels.

Second, the distribution of osteoporotic cases differed among vertebral levels, with the lowest proportion at L3 and the highest proportion at L2. This class imbalance may have influenced the comparison of model performance across levels.

Third, lumbar spine DEXA may overestimate BMD in the presence of degenerative endplate sclerosis, osteophytes, facet arthropathy, aortic calcification, and other degenerative changes. These factors were not systematically quantified or incorporated into the reference-standard interpretation in the present study. Consequently, some patients may have been misclassified because of falsely elevated lumbar DEXA measurements.

Although all eligible CT examinations were directly reviewed, subtle or asymptomatic chronic vertebral compression fractures may have been overlooked, particularly when vertebral deformity was mild. Therefore, some degree of residual misclassification cannot be excluded.

The study also included both contrast-enhanced and non-contrast CT examinations. Although previous attenuation-based studies have suggested that the effect of contrast on conventional HU measurements may be limited [3,25,26], radiomic features may be more sensitive to contrast administration and other acquisition-related factors. This concern is supported by dedicated radiomic feature stability studies: in a combined phantom and clinical-cohort analysis, Jha et al. found that the presence or absence of intravenous contrast medium had a stronger adverse effect on radiomic feature reproducibility than differences in slice thickness, with only 14% of features showing good reproducibility between contrast-enhanced and non-contrast acquisitions, compared with 47% across different slice thicknesses [27]. To our knowledge, no study has specifically examined radiomic feature stability under contrast administration in vertebral bone; we therefore draw on findings from other anatomical regions, where the underlying feature-extraction methodology is the same.

Finally, this was a single-center study without external validation. All CT examinations were obtained using a single scanner and a standardized protocol, which reduced inter-scanner and inter-protocol heterogeneity but may limit generalizability to other scanners, institutions, and populations [28].

Building on these findings and the limitations outlined above, future research should prioritize external validation using independent, multicenter cohorts acquired on different CT scanners and protocols; development of patient-level, multivertebral models based on combined L1–L4 DEXA classification rather than single-vertebra analysis; dedicated subgroup analyses or harmonization strategies to evaluate the effect of IVCM on radiomic features; systematic quantification of degenerative changes affecting DEXA accuracy; evaluation of broader and more stable radiomic feature sets across acquisition conditions; and the use of class imbalance-aware metrics, such as balanced Acc or macro-averaged F1-score, in larger and more balanced datasets.

## 5. Conclusions

We developed and internally validated CT radiomics-based ML models for the three-class differentiation of normal BMD, osteopenia, and osteoporosis in adults aged over 50 years. Classification performance varied across lumbar vertebral levels: L3 demonstrated the most favorable overall performance, L2 showed the strongest results for osteoporosis discrimination, and LR was generally the best-performing algorithm, whereas osteopenia remained the most challenging category to classify and L1 showed a comparatively weaker performance. These findings highlight meaningful level-dependent differences in vertebral radiomic information but should be interpreted as exploratory rather than as support for clinical BMD classification from a single lumbar vertebra. The proposed approach may serve as an opportunistic screening and triage tool to identify patients who could benefit from formal DEXA and further osteoporosis evaluation. Patient-level multivertebral modeling, systematic assessment of degenerative factors affecting DEXA, and external multicenter validation are required before clinical implementation.

## Figures and Tables

**Figure 1:**
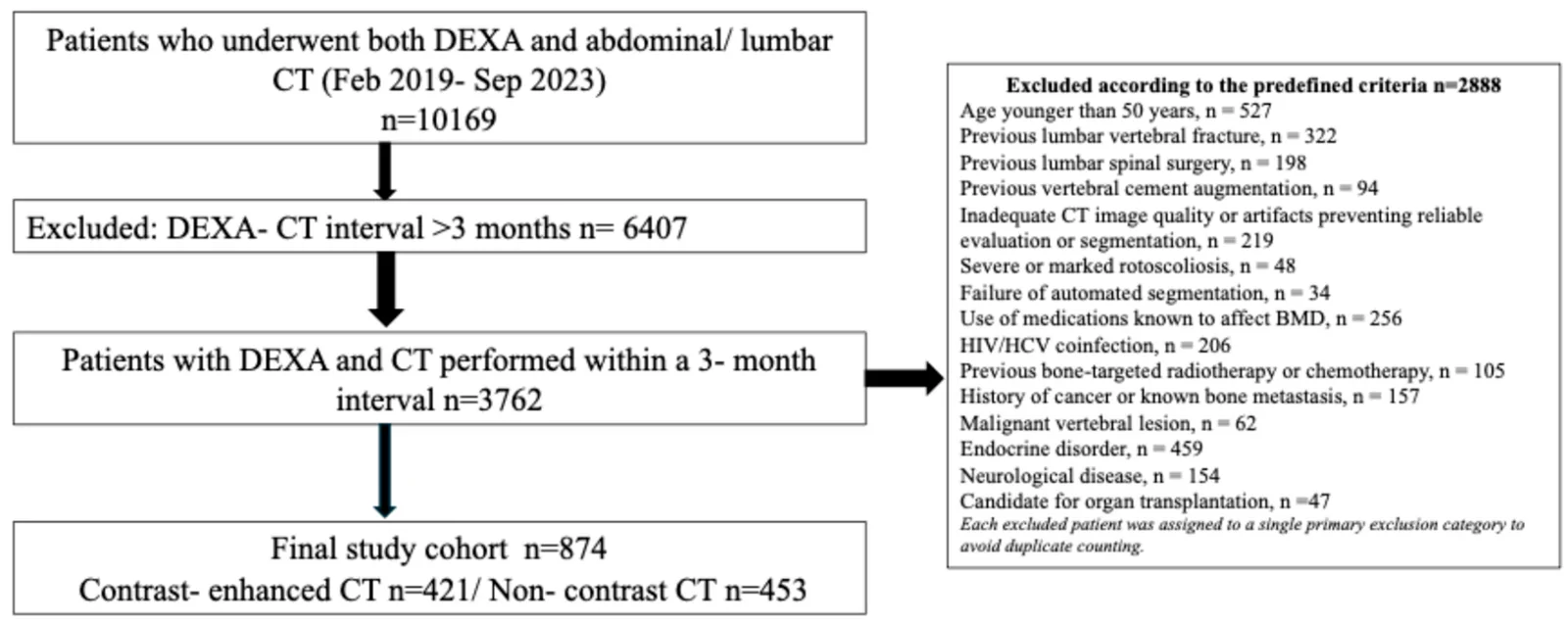
Flow diagram illustrating patient selection process.

**Figure 2:**
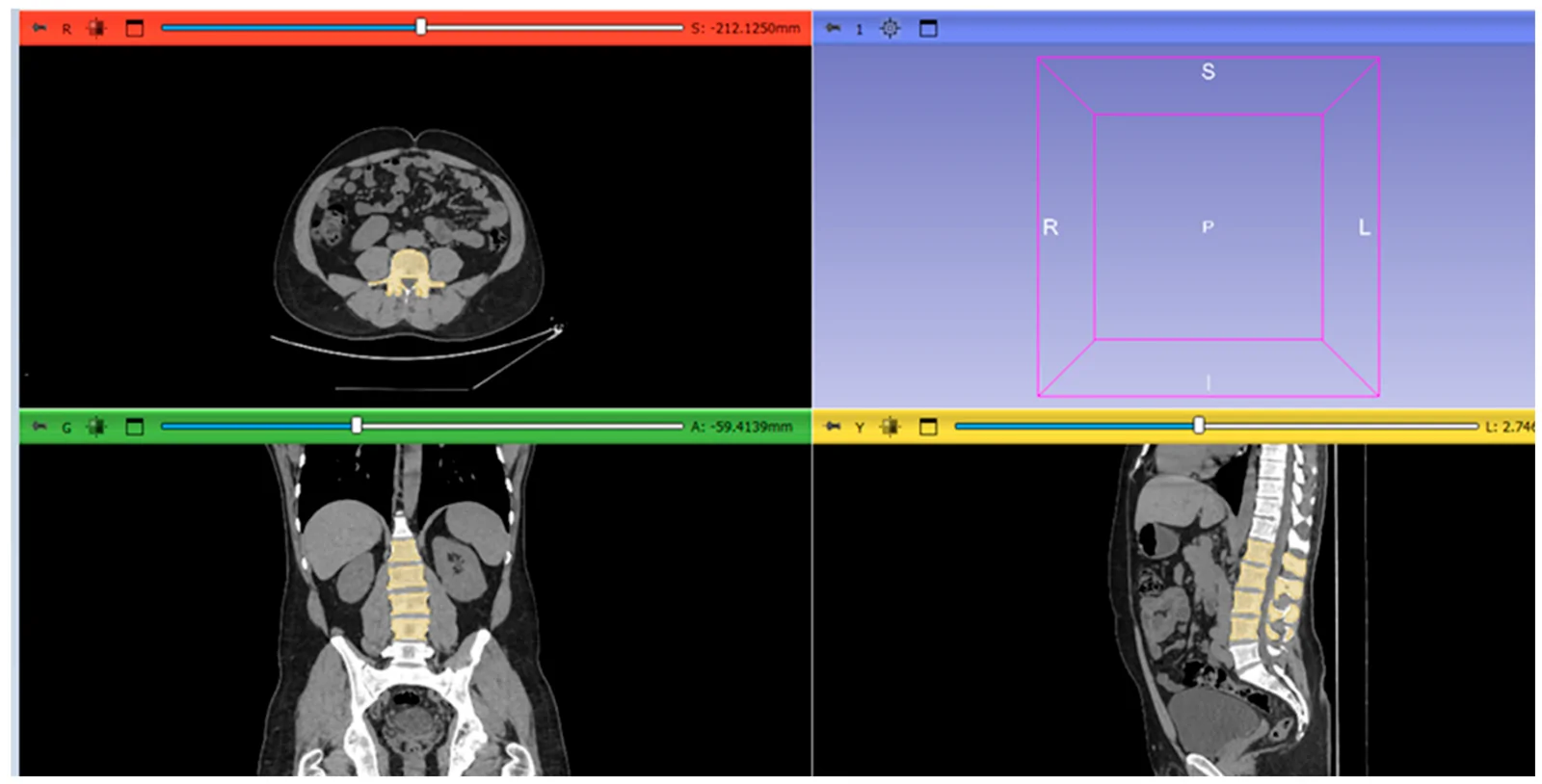
Automated segmentation of lumbar vertebrae using the 3D Slicer platform.

**Figure 3:**
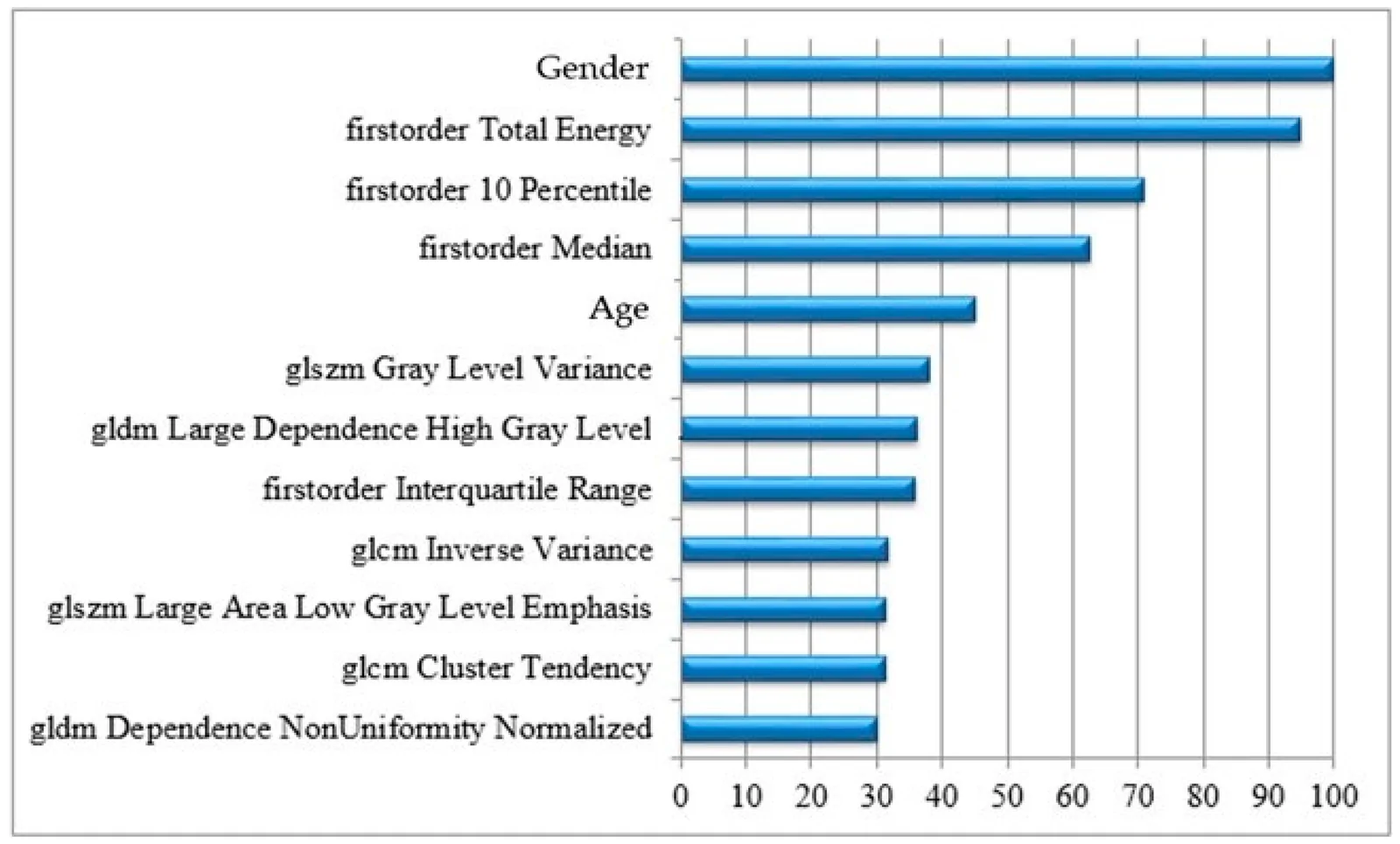
Results of Variable Importance Based on the Outcome Variable for L4 vertebra.

**Figure 4:**
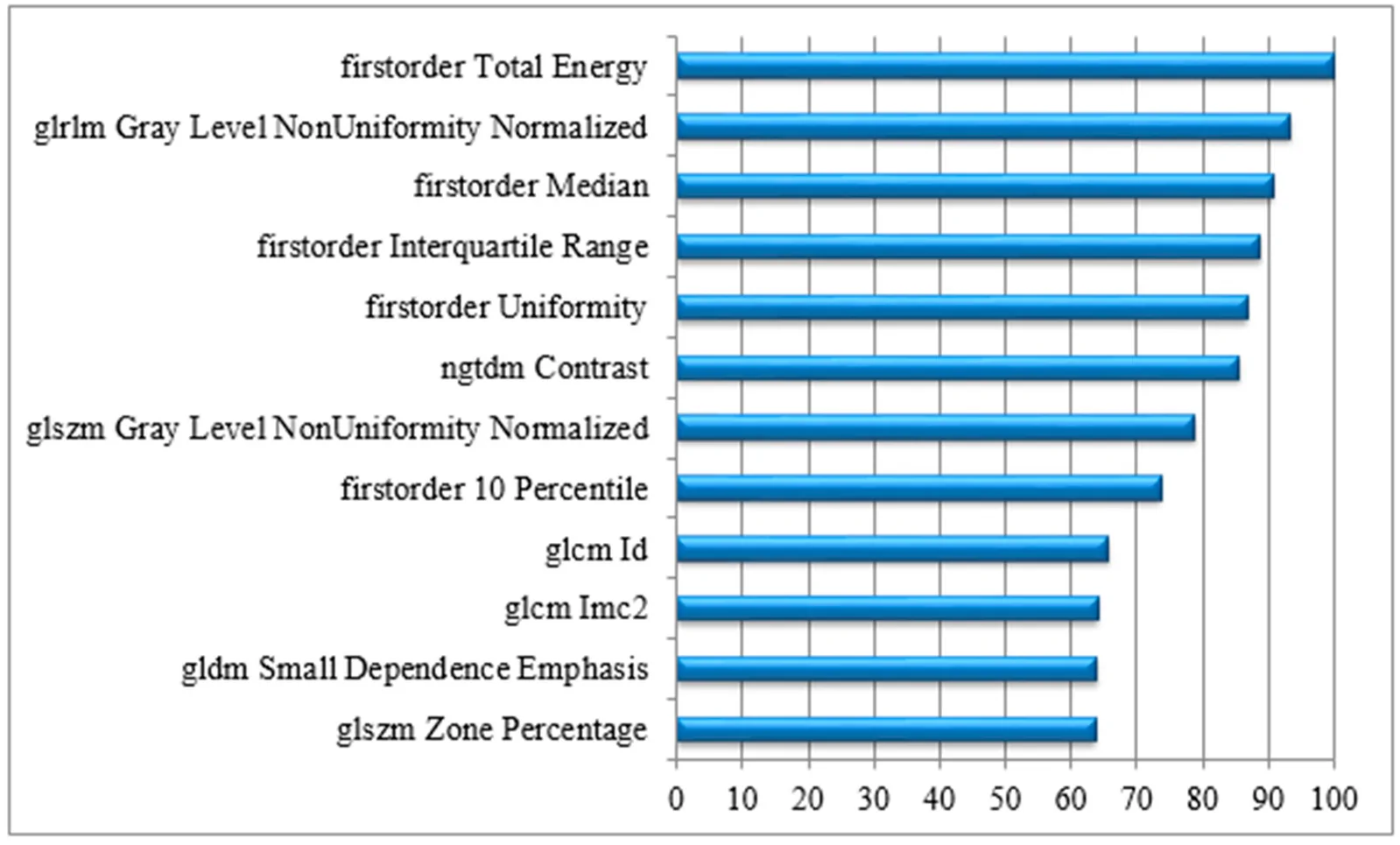
Results of Variable Importance Based on the Outcome Variable for L3 vertebra.

**Figure 5:**
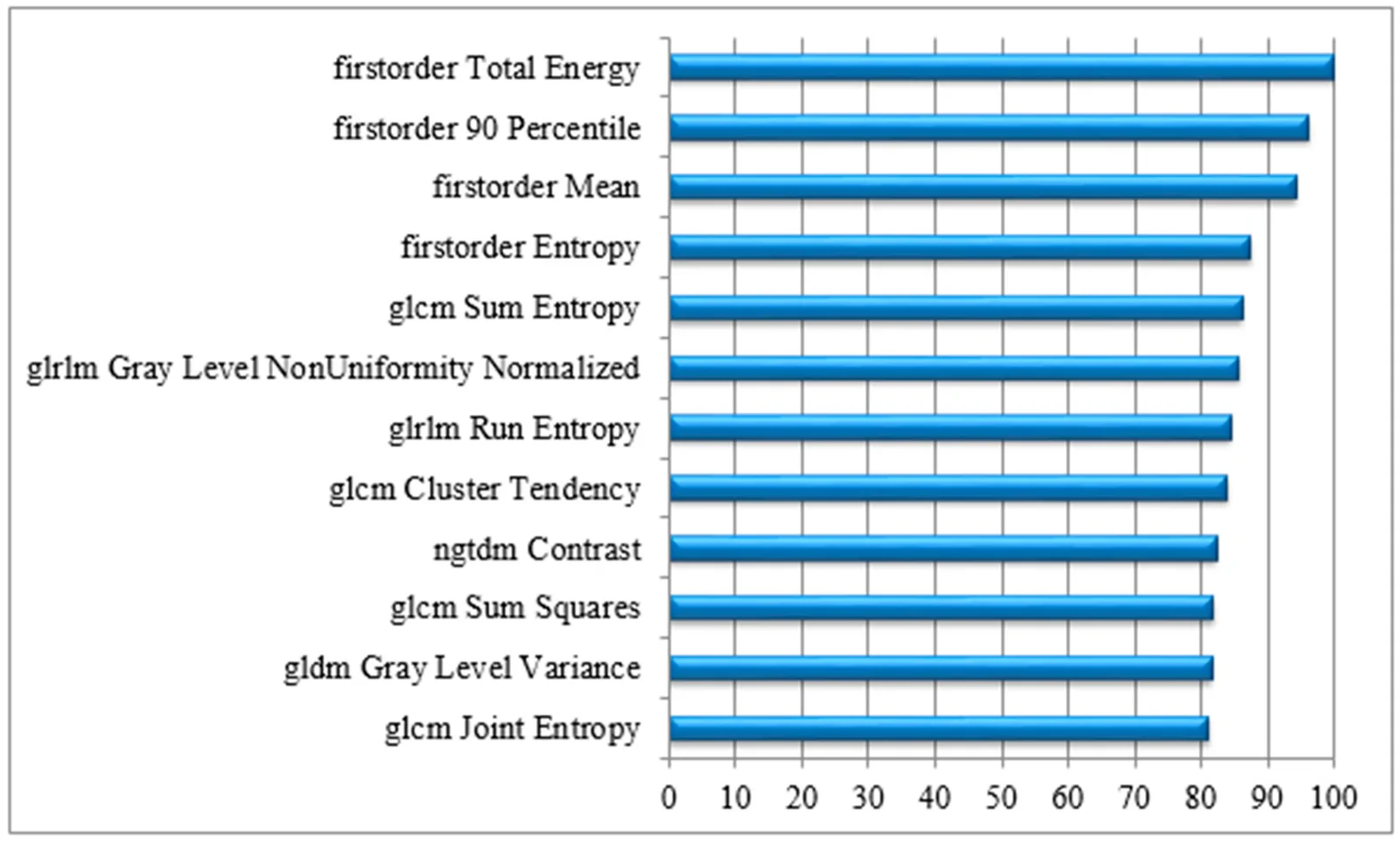
Results of Variable Importance Based on the Outcome Variable for L2 vertebra.

**Figure 6:**
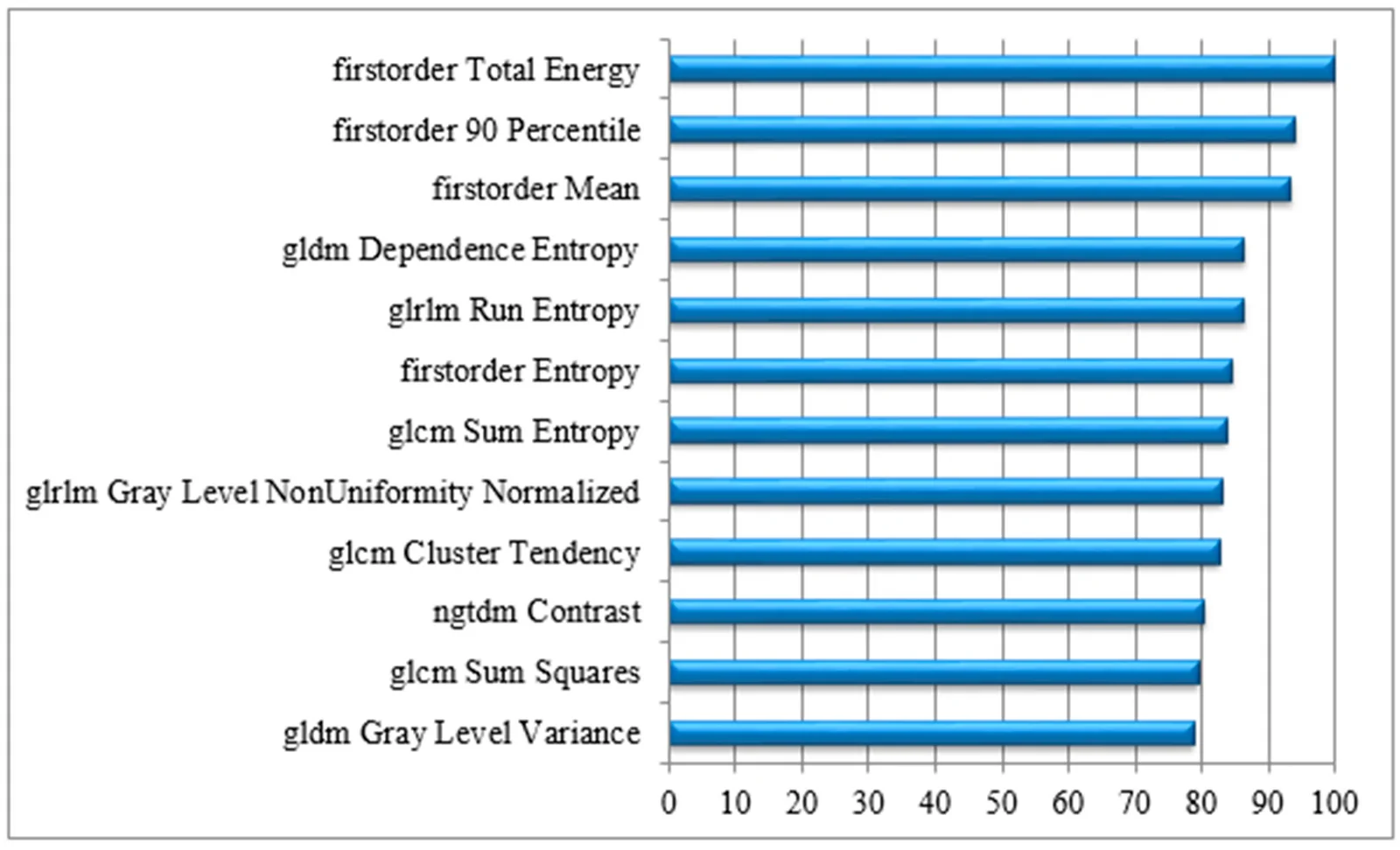
Results of Variable Importance Based on the Outcome Variable for L1 vertebra.

**Table 1:** Demographic Data.

Variable		
Age (years)	Mean ± SD	64.36 ± 9.73
	Median (Min–Max)	63.00 (50.00–95.00)
Sex, n (%)	Female	674 (77.1)
	Male	200 (22.9)

Abbreviations: SD = standard deviation; Min = minimum; Max = maximum.

**Table 2:** Distribution of Bone Mineral Density (BMD) Classification According to T-Scores at Each Lumbar Vertebral Level.

Variable	L4 n (%)	L3 n (%)	L2 n (%)	L1 n (%)	*p* Value
Classification by T-score					
Normal	369 (42.3)	382 (43.7)	277 (31.7)	282 (32.3)	<0.001 ^a^
Osteopenia	326 (37.3)	353 (40.4)	332 (38.0)	402 (46.0)	
Osteoporosis	179 (20.4)	139 (15.9)	265 (30.3)	190 (21.7)	
Total	874 (100.0)	874 (100.0)	874 (100.0)	874 (100.0)	

Abbreviations: BMD = bone mineral density. ^a^ Chi-square test.

**Table 3:** Machine Learning Performance Metrics for L4 vertebra.

Methods		Acc	F-Score	ROC Area	PRC Area
Logistic Regression (LR)	Normal	0.743	0.745	0.868	0.828
	Osteopenia	0.601	0.567	0.719	0.524
	Osteoporosis	0.500	0.558	0.876	0.668
	General	0.640	0.640	0.814	0.682
Multilayer Perceptron (MLP)	Normal	0.762	0.745	0.860	0.803
	Osteopenia	0.577	0.550	0.688	0.513
	Osteoporosis	0.455	0.526	0.866	0.638
	General	0.630	0.628	0.797	0.661
Bagging	Normal	0.743	0.745	0.861	0.822
	Osteopenia	0.558	0.543	0.688	0.499
	Osteoporosis	0.539	0.565	0.869	0.633
	General	0.632	0.633	0.798	0.663
Decision Table (DT)	Normal	0.661	0.716	0.855	0.813
	Osteopenia	0.699	0.594	0.703	0.531
	Osteoporosis	0.427	0.514	0.859	0.626
	General	0.628	0.629	0.799	0.670
Random Forest (RF)	Normal	0.734	0.745	0.872	0.837
	Osteopenia	0.604	0.565	0.701	0.504
	Osteoporosis	0.522	0.579	0.877	0.653
	General	0.643	0.644	0.809	0.675

Abbreviations: Acc = accuracy; F-score = harmonic mean of precision and recall; ROC area = area under the receiver operating characteristic curve; PRC area = area under the precision–recall curve.

**Table 4:** Machine Learning Performance Metrics for L3 vertebra.

Methods		Acc	F-Score	ROC Area	PRC Area
Logistic Regression (LR)	Normal	0.804	0.809	0.912	0.892
	Osteopenia	0.722	0.675	0.789	0.667
	Osteoporosis	0.460	0.547	0.891	0.640
	General	0.716	0.713	0.859	0.761
Multilayer Perceptron (MLP)	Normal	0.835	0.812	0.905	0.864
	Osteopenia	0.669	0.646	0.761	0.616
	Osteoporosis	0.424	0.511	0.862	0.585
	General	0.703	0.697	0.840	0.720
Bagging	Normal	0.798	0.789	0.894	0.869
	Osteopenia	0.652	0.625	0.741	0.588
	Osteoporosis	0.410	0.477	0.867	0.539
	General	0.677	0.673	0.828	0.703
Decision Table (DT)	Normal	0.796	0.775	0.881	0.849
	Osteopenia	0.654	0.631	0.750	0.594
	Osteoporosis	0.424	0.511	0.850	0.551
	General	0.680	0.675	0.823	0.699
Random Forest (RF)	Normal	0.822	0.803	0.900	0.874
	Osteopenia	0.686	0.655	0.749	0.590
	Osteoporosis	0.410	0.502	0.866	0.589
	General	0.701	0.695	0.834	0.714

Abbreviations: Acc = accuracy; F-score = harmonic mean of precision and recall; ROC area = area under the receiver operating characteristic curve; PRC area = area under the precision–recall curve.

**Table 5:** Machine Learning Performance Metrics for L2 vertebra.

Methods		Acc	F-Score	ROC Area	PRC Area
Logistic Regression (LR)	Normal	0.758	0.779	0.927	0.869
	Osteopenia	0.642	0.627	0.766	0.599
	Osteoporosis	0.725	0.725	0.897	0.797
	General	0.704	0.705	0.857	0.745
Multilayer Perceptron (MLP)	Normal	0.751	0.755	0.915	0.845
	Osteopenia	0.617	0.605	0.750	0.581
	Osteoporosis	0.702	0.717	0.889	0.779
	General	0.685	0.686	0.844	0.725
Bagging	Normal	0.762	0.748	0.908	0.821
	Osteopenia	0.563	0.562	0.706	0.556
	Osteoporosis	0.653	0.668	0.869	0.739
	General	0.653	0.653	0.819	0.695
Decision Table (DT)	Normal	0.733	0.740	0.900	0.807
	Osteopenia	0.660	0.598	0.693	0.516
	Osteoporosis	0.543	0.618	0.855	0.703
	General	0.648	0.649	0.808	0.665
Random Forest (RF)	Normal	0.740	0.748	0.910	0.828
	Osteopenia	0.569	0.558	0.697	0.543
	Osteoporosis	0.634	0.642	0.866	0.723
	General	0.643	0.644	0.816	0.688

Abbreviations: Acc = accuracy; F-score = harmonic mean of precision and recall; ROC area = area under the receiver operating characteristic curve; PRC area = area under the precision–recall curve.

**Table 6:** Machine Learning Performance Metrics for L1 vertebra.

Methods		Acc	F-Score	ROC Area	PRC Area
Logistic Regression (LR)	Normal	0.709	0.752	0.914	0.843
	Osteopenia	0.754	0.688	0.733	0.621
	Osteoporosis	0.484	0.549	0.867	0.632
	General	0.681	0.678	0.820	0.695
Multilayer Perceptron (MLP)	Normal	0.702	0.740	0.901	0.823
	Osteopenia	0.677	0.646	0.708	0.595
	Osteoporosis	0.537	0.550	0.850	0.599
	General	0.654	0.656	0.801	0.669
Bagging	Normal	0.702	0.716	0.891	0.813
	Osteopenia	0.669	0.627	0.693	0.599
	Osteoporosis	0.426	0.481	0.847	0.615
	General	0.627	0.624	0.791	0.671
Decision Table (DT)	Normal	0.699	0.700	0.880	0.779
	Osteopenia	0.677	0.635	0.703	0.626
	Osteoporosis	0.442	0.512	0.835	0.578
	General	0.633	0.629	0.789	0.665
Random Forest (RF)	Normal	0.684	0.708	0.885	0.800
	Osteopenia	0.692	0.636	0.675	0.561
	Osteoporosis	0.426	0.492	0.845	0.601
	General	0.632	0.628	0.780	0.647

Abbreviations: Acc = accuracy; F-score = harmonic mean of precision and recall; ROC area = area under the receiver operating characteristic curve; PRC area = area under the precision–recall curve.

**Table 7:** Lowest and highest ML model performance results at all vertebral levels.

Performance Metrics		Lowest		Highest	
		Vertebra Level/Model Name	Model Value	Vertebra Level/Model Name	Model Value
Accuracy	Normal	L4/DT	66.1%	L3/MLP	83.5%
	Osteopenia	L4/Bagging	55.8%	L1/LR	75.4%
	Osteoporosis	L3/RF and Bagging	41%	L2/LR	72.5%
	General	L1/Bagging	62.7%	L3/LR	71.6%
F-score	Normal	L1/DT	70%	L3/MLP	81.2%
	Osteopenia	L4/Bagging	54.3%	L1/LR	68.8%
	Osteoporosis	L3/Bagging	47.7%	L2/LR	72.5%
	General	L1/Bagging	62.3%	L3/LR	71.3%
ROC area	Normal	L4/DT	0.855	L2/LR	0.927
	Osteopenia	L1/RF	0.675	L3/LR	0.789
	Osteoporosis	L1/DT	0.835	L2/LR	0.897
	General	L1/RF	0.780	L3/LR	0.859
PRC area	Normal	L1/DT	0.779	L3/LR	0.892
	Osteopenia	L4/Bagging	0.499	L3/LR	0.667
	Osteoporosis	L3/Bagging	0.539	L2/LR	0.797
	General	L1/DT	0.647	L3/LR	0.761

Abbreviations: PRC: Precision–Recall Curve, DT: Decision Table, RF: Random Forest, MLP: Multilayer Perceptron, LR: Logistic Regression.

## Data Availability

The datasets generated and analyzed during the current study are not publicly available because they contain sensitive clinical and medical imaging data and are subject to patient privacy, ethical, and institutional data protection restrictions. Requests for further information may be directed to the corresponding author.

## Supplementary Materials

The following supporting information can be downloaded at: https://www.mdpi.com/article/10.3390/tomography12100147/s1. Table S1: Complete list of the 93 radiomic features extracted from each lumbar vertebra (L1–L4) using the PyRadiomics library, itemized by feature category: first-order (n = 18), gray level co-occurrence matrix (GLCM, n = 24), gray level dependence matrix (GLDM, n = 14), gray level run length matrix (GLRLM, n = 16), gray level size zone matrix (GLSZM, n = 16), and neighborhood gray tone difference matrix (NGTDM, n = 5); Table S2: Reproducibility check: mean ± standard deviation (SD) of model performance metrics from an independent reanalysis of the underlying patient-level dataset, performed as a robustness check for the point estimates reported in Table 3, Table 4, Table 5 and Table 6 of the main text.
