# Supplementary material for: CT Radiomics and Machine Learning for Three-Class Bone Density Classification: An Exploratory Comparison Across Lumbar Vertebral Levels
Source: Tomography. 2026 Oct 2;12(10):147. doi: 10.3390/tomography12100147 (PMC13709906; doi:10.3390/tomography12100147)
Supplement: Supplementary file 1 [file tomography-12-00147-s001.zip › tomography-4485836-supplementary.pdf]

Supplementary Table S1

Complete list of the 93 radiomic features extracted from each lumbar vertebra (L1–L4) using the PyRadiomics library, itemized by feature category: first-order (n = 18), gray level co-occurrence matrix (GLCM, n = 24), gray level dependence matrix (GLDM, n = 14), gray level run length matrix (GLRLM, n = 16), gray level size zone matrix (GLSZM, n = 16), and neighborhood gray tone difference matrix (NGTDM, n = 5).

Feature Category	Feature Name (PyRadiomics identifier)
**First-order (18)**	firstorder_10Percentile
	firstorder_90Percentile
	firstorder_Energy
	firstorder_Entropy
	firstorder_InterquartileRange
	firstorder_Kurtosis
	firstorder_Maximum
	firstorder_MeanAbsoluteDeviation
	firstorder_Mean
	firstorder_Median
	firstorder_Minimum
	firstorder_Range
	firstorder_RobustMeanAbsoluteDeviation
	firstorder_RootMeanSquared
	firstorder_Skewness
	firstorder_TotalEnergy
	firstorder_Uniformity
	firstorder_Variance
**GLCM (24)**	glcm_Autocorrelation
	glcm_ClusterProminence
	glcm_ClusterShade
	glcm_ClusterTendency
	glcm_Contrast
	glcm_Correlation
	glcm_DifferenceAverage
	glcm_DifferenceEntropy
	glcm_DifferenceVariance
	glcm_Id
	glcm_Idm
	glcm_Idmn
	glcm_Idn
	glcm_Imc1
	glcm_Imc2
	glcm_InverseVariance
	glcm_JointAverage
	glcm_JointEnergy
	glcm_JointEntropy
	glcm_MCC
	glcm_MaximumProbability
	glcm_SumAverage

Feature Category	Feature Name (PyRadiomics identifier)
	glcm_SumEntropy
	glcm_SumSquares
**GLDM (14)**	gldm_DependenceEntropy
	gldm_DependenceNonUniformity
	gldm_DependenceNonUniformityNormalized
	gldm_DependenceVariance
	gldm_GrayLevelNonUniformity
	gldm_GrayLevelVariance
	gldm_HighGrayLevelEmphasis
	gldm_LargeDependenceEmphasis
	gldm_LargeDependenceHighGrayLevelEmphasis
	gldm_LargeDependenceLowGrayLevelEmphasis
	gldm_LowGrayLevelEmphasis
	gldm_SmallDependenceEmphasis
	gldm_SmallDependenceHighGrayLevelEmphasis
	gldm_SmallDependenceLowGrayLevelEmphasis
**GLRLM (16)**	glrlm_GrayLevelNonUniformity
	glrlm_GrayLevelNonUniformityNormalized
	glrlm_GrayLevelVariance
	glrlm_HighGrayLevelRunEmphasis
	glrlm_LongRunEmphasis
	glrlm_LongRunHighGrayLevelEmphasis
	glrlm_LongRunLowGrayLevelEmphasis
	glrlm_LowGrayLevelRunEmphasis
	glrlm_RunEntropy
	glrlm_RunLengthNonUniformity
	glrlm_RunLengthNonUniformityNormalized
	glrlm_RunPercentage
	glrlm_RunVariance
	glrlm_ShortRunEmphasis
	glrlm_ShortRunHighGrayLevelEmphasis
	glrlm_ShortRunLowGrayLevelEmphasis
**GLSZM (16)**	glszm_GrayLevelNonUniformity
	glszm_GrayLevelNonUniformityNormalized
	glszm_GrayLevelVariance
	glszm_HighGrayLevelZoneEmphasis
	glszm_LargeAreaEmphasis
	glszm_LargeAreaHighGrayLevelEmphasis
	glszm_LargeAreaLowGrayLevelEmphasis
	glszm_LowGrayLevelZoneEmphasis
	glszm_SizeZoneNonUniformity
	glszm_SizeZoneNonUniformityNormalized
	glszm_SmallAreaEmphasis
	glszm_SmallAreaHighGrayLevelEmphasis
	glszm_SmallAreaLowGrayLevelEmphasis
	glszm_ZoneEntropy
	glszm_ZonePercentage
	glszm_ZoneVariance

Feature Category	Feature Name (PyRadiomics identifier)
**NGTDM (5)**	ngtdm_Busyness
	ngtdm_Coarseness
	ngtdm_Complexity
	ngtdm_Contrast
	ngtdm_Strength

Supplementary Table S2

Reproducibility check: mean ± standard deviation (SD) of model performance metrics from an independent reanalysis of the underlying patient-level dataset, performed as a robustness check for the point estimates reported in Tables 3–6 of the main text.

Methodology note: The original analysis (reported in the main text) was performed in R using the RWeka package, with repeated stratified 10-fold cross-validation across 1,000 repetitions. Because the repetition-level outputs of that original analysis were not retained, we performed an independent reanalysis of the patient-level radiomic feature dataset (874 patients × 4 vertebral levels × 93 features) using Python (scikit-learn), following the same overall workflow described in Section 2.6: correlation-based feature selection performed within each training fold only, the same five classifiers, and stratified 10-fold cross-validation. Due to the computational cost of this post hoc reanalysis, 100 repetitions (1,000 folds per vertebral level) were performed rather than 1,000. The resulting point estimates are close to, though not numerically identical to, those reported in Tables 3–6, which is expected given the different software implementation (Python/scikit-learn vs. R/RWeka); the relative pattern across vertebral levels and classifiers — including the finding that L3 shows the strongest overall performance — is preserved. *Weka's DecisionTable classifier has no exact scikit-learn equivalent; a decision-tree classifier was used as the closest available approximation for this reproducibility check only.

Vertebral level: L4

Method	Class	Accuracy	F-score	ROC area	PRC area
**Logistic Regression (LR)**	Normal	0.781 ± 0.068	0.779 ± 0.048	0.890 ± 0.033	0.866 ± 0.043
	Osteopenia	0.626 ± 0.085	0.596 ± 0.063	0.737 ± 0.050	0.574 ± 0.070
	Osteoporosis	0.521 ± 0.114	0.571 ± 0.097	0.882 ± 0.041	0.682 ± 0.090
	**General**	0.670 ± 0.047	0.668 ± 0.048	0.831 ± 0.032	0.719 ± 0.045
**Multilayer Perceptron (MLP)**	Normal	0.750 ± 0.107	0.728 ± 0.071	0.853 ± 0.053	0.825 ± 0.065
	Osteopenia	0.450 ± 0.189	0.456 ± 0.147	0.668 ± 0.070	0.509 ± 0.071
	Osteoporosis	0.523 ± 0.186	0.489 ± 0.133	0.818 ± 0.083	0.583 ± 0.124
	**General**	0.592 ± 0.064	0.578 ± 0.079	0.777 ± 0.053	0.657 ± 0.060
**Bagging**	Normal	0.752 ± 0.071	0.762 ± 0.050	0.868 ± 0.037	0.836 ± 0.049
	Osteopenia	0.584 ± 0.088	0.555 ± 0.064	0.700 ± 0.051	0.533 ± 0.061
	Osteoporosis	0.492 ± 0.116	0.520 ± 0.098	0.851 ± 0.046	0.604 ± 0.094
	**General**	0.636 ± 0.047	0.635 ± 0.047	0.802 ± 0.034	0.675 ± 0.045
**Decision Table (DT)***	Normal	0.679 ± 0.081	0.683 ± 0.059	0.727 ± 0.047	0.607 ± 0.052
	Osteopenia	0.478 ± 0.087	0.475 ± 0.070	0.582 ± 0.052	0.426 ± 0.039
	Osteoporosis	0.476 ± 0.124	0.469 ± 0.101	0.668 ± 0.064	0.340 ± 0.072
	**General**	0.563 ± 0.051	0.561 ± 0.051	0.661 ± 0.040	0.485 ± 0.038
**Random Forest (RF)**	Normal	0.751 ± 0.071	0.766 ± 0.050	0.876 ± 0.035	0.847 ± 0.048
	Osteopenia	0.611 ± 0.084	0.571 ± 0.061	0.707 ± 0.049	0.535 ± 0.060
	Osteoporosis	0.483 ± 0.116	0.521 ± 0.100	0.858 ± 0.044	0.624 ± 0.092
	**General**	0.644 ± 0.047	0.643 ± 0.047	0.809 ± 0.033	0.685 ± 0.044

Vertebral level: L3

Method	Class	Accuracy	F-score	ROC area	PRC area
**Logistic Regression (LR)**	Normal	0.789 ± 0.064	0.798 ± 0.047	0.910 ± 0.029	0.894 ± 0.036
	Osteopenia	0.737 ± 0.074	0.676 ± 0.054	0.784 ± 0.047	0.678 ± 0.070
	Osteoporosis	0.427 ± 0.130	0.522 ± 0.125	0.887 ± 0.042	0.653 ± 0.110
	**General**	0.710 ± 0.047	0.705 ± 0.049	0.856 ± 0.032	0.768 ± 0.047
**Multilayer Perceptron (MLP)**	Normal	0.789 ± 0.091	0.776 ± 0.059	0.888 ± 0.045	0.872 ± 0.050
	Osteopenia	0.618 ± 0.157	0.588 ± 0.114	0.722 ± 0.057	0.588 ± 0.070
	Osteoporosis	0.320 ± 0.173	0.355 ± 0.158	0.804 ± 0.095	0.504 ± 0.141
	**General**	0.645 ± 0.066	0.633 ± 0.071	0.807 ± 0.047	0.698 ± 0.057
**Bagging**	Normal	0.798 ± 0.063	0.788 ± 0.046	0.890 ± 0.033	0.859 ± 0.046
	Osteopenia	0.667 ± 0.080	0.634 ± 0.058	0.740 ± 0.050	0.608 ± 0.064
	Osteoporosis	0.402 ± 0.134	0.471 ± 0.128	0.850 ± 0.052	0.576 ± 0.112
	**General**	0.682 ± 0.048	0.676 ± 0.050	0.823 ± 0.035	0.713 ± 0.049
**Decision Table (DT)***	Normal	0.715 ± 0.074	0.717 ± 0.054	0.750 ± 0.046	0.643 ± 0.052
	Osteopenia	0.532 ± 0.086	0.530 ± 0.066	0.607 ± 0.051	0.475 ± 0.041

Method	Class	Accuracy	F-score	ROC area	PRC area
	Osteoporosis	0.428 ± 0.138	0.420 ± 0.115	0.658 ± 0.070	0.283 ± 0.079
	**General**	0.595 ± 0.050	0.594 ± 0.050	0.677 ± 0.041	0.518 ± 0.040
**Random Forest (RF)**	Normal	0.802 ± 0.062	0.792 ± 0.046	0.897 ± 0.032	0.871 ± 0.045
	Osteopenia	0.685 ± 0.080	0.645 ± 0.057	0.749 ± 0.048	0.616 ± 0.064
	Osteoporosis	0.385 ± 0.132	0.467 ± 0.130	0.860 ± 0.049	0.597 ± 0.112
	**General**	0.689 ± 0.047	0.681 ± 0.049	0.831 ± 0.034	0.725 ± 0.048

Vertebral level: L2

Method	Class	Accuracy	F-score	ROC area	PRC area
**Logistic Regression (LR)**	Normal	0.757 ± 0.082	0.780 ± 0.058	0.925 ± 0.027	0.864 ± 0.048
	Osteopenia	0.642 ± 0.082	0.617 ± 0.062	0.750 ± 0.050	0.614 ± 0.070
	Osteoporosis	0.684 ± 0.089	0.695 ± 0.065	0.884 ± 0.033	0.782 ± 0.063
	**General**	0.692 ± 0.048	0.692 ± 0.048	0.846 ± 0.030	0.744 ± 0.046
**Multilayer Perceptron (MLP)**	Normal	0.788 ± 0.125	0.728 ± 0.094	0.898 ± 0.067	0.825 ± 0.083
	Osteopenia	0.397 ± 0.183	0.433 ± 0.159	0.676 ± 0.070	0.527 ± 0.074
	Osteoporosis	0.685 ± 0.140	0.629 ± 0.091	0.841 ± 0.062	0.716 ± 0.087
	**General**	0.608 ± 0.065	0.586 ± 0.087	0.796 ± 0.054	0.678 ± 0.064
**Bagging**	Normal	0.737 ± 0.082	0.732 ± 0.058	0.906 ± 0.031	0.822 ± 0.057
	Osteopenia	0.545 ± 0.085	0.537 ± 0.066	0.691 ± 0.053	0.566 ± 0.065
	Osteoporosis	0.628 ± 0.096	0.639 ± 0.072	0.860 ± 0.036	0.708 ± 0.071
	**General**	0.631 ± 0.049	0.630 ± 0.049	0.811 ± 0.032	0.690 ± 0.047
**Decision Table (DT)***	Normal	0.662 ± 0.089	0.667 ± 0.067	0.757 ± 0.048	0.558 ± 0.065
	Osteopenia	0.488 ± 0.089	0.484 ± 0.071	0.585 ± 0.053	0.435 ± 0.040
	Osteoporosis	0.584 ± 0.095	0.580 ± 0.074	0.700 ± 0.053	0.470 ± 0.062
	**General**	0.572 ± 0.052	0.571 ± 0.052	0.674 ± 0.040	0.485 ± 0.041
**Random Forest (RF)**	Normal	0.746 ± 0.082	0.746 ± 0.058	0.913 ± 0.029	0.836 ± 0.053
	Osteopenia	0.568 ± 0.085	0.553 ± 0.064	0.699 ± 0.052	0.572 ± 0.065
	Osteoporosis	0.632 ± 0.096	0.648 ± 0.070	0.867 ± 0.034	0.726 ± 0.068
	**General**	0.644 ± 0.047	0.643 ± 0.047	0.818 ± 0.031	0.702 ± 0.046

Vertebral level: L1

Method	Class	Accuracy	F-score	ROC area	PRC area
**Logistic Regression (LR)**	Normal	0.725 ± 0.083	0.750 ± 0.062	0.913 ± 0.029	0.845 ± 0.052
	Osteopenia	0.752 ± 0.068	0.688 ± 0.047	0.745 ± 0.051	0.663 ± 0.065
	Osteoporosis	0.462 ± 0.114	0.534 ± 0.105	0.867 ± 0.042	0.673 ± 0.093
	**General**	0.680 ± 0.047	0.675 ± 0.049	0.826 ± 0.034	0.724 ± 0.051
**Multilayer Perceptron (MLP)**	Normal	0.760 ± 0.108	0.732 ± 0.073	0.896 ± 0.044	0.821 ± 0.071
	Osteopenia	0.610 ± 0.152	0.603 ± 0.111	0.692 ± 0.063	0.613 ± 0.069
	Osteoporosis	0.496 ± 0.163	0.509 ± 0.128	0.834 ± 0.072	0.623 ± 0.116
	**General**	0.633 ± 0.065	0.624 ± 0.075	0.789 ± 0.048	0.683 ± 0.061
**Bagging**	Normal	0.720 ± 0.084	0.733 ± 0.061	0.894 ± 0.033	0.812 ± 0.060
	Osteopenia	0.697 ± 0.075	0.648 ± 0.051	0.705 ± 0.052	0.626 ± 0.059
	Osteoporosis	0.440 ± 0.113	0.501 ± 0.101	0.856 ± 0.043	0.632 ± 0.089
	**General**	0.649 ± 0.048	0.643 ± 0.049	0.799 ± 0.036	0.688 ± 0.050
**Decision Table (DT)***	Normal	0.668 ± 0.087	0.660 ± 0.064	0.750 ± 0.048	0.550 ± 0.062

Method	Class	Accuracy	F-score	ROC area	PRC area
	Osteopenia	0.558 ± 0.082	0.560 ± 0.062	0.596 ± 0.051	0.521 ± 0.037
	Osteoporosis	0.491 ± 0.119	0.489 ± 0.097	0.676 ± 0.062	0.362 ± 0.071
	**General**	0.579 ± 0.051	0.577 ± 0.052	0.663 ± 0.041	0.496 ± 0.041
**Random Forest (RF)**	Normal	0.719 ± 0.084	0.740 ± 0.063	0.900 ± 0.033	0.823 ± 0.060
	Osteopenia	0.718 ± 0.072	0.658 ± 0.050	0.706 ± 0.053	0.623 ± 0.060
	Osteoporosis	0.428 ± 0.115	0.494 ± 0.104	0.858 ± 0.041	0.642 ± 0.087
	**General**	0.655 ± 0.049	0.649 ± 0.050	0.802 ± 0.037	0.692 ± 0.050
